# The Psychology of Food Cravings in Patients With First-Episode Psychosis

**DOI:** 10.3389/fpsyt.2020.587486

**Published:** 2020-12-11

**Authors:** Young-Hyuk Kim, Seunghyong Ryu, Hee-Jung Nam, Mina Kim, Min Jhon, Ju-Yeon Lee, Jae-Min Kim, Min Ho Shin, Young-Chul Chung, Sung-Wan Kim

**Affiliations:** ^1^Department of Psychiatry, Chonnam National University Medical School, Gwangju, South Korea; ^2^Department of Psychiatry, Seoul Medical Center, Seoul, South Korea; ^3^Gwangju Mental Health and Welfare Commission, Gwangju, South Korea; ^4^Department of Nursing, Graduate School, Chonnam National University, Gwangju, South Korea; ^5^Department of Preventive Medicine, Chonnam National University Medical School, Hwasun, South Korea; ^6^Department of Psychiatry, Chonbuk National University Medical School, Jeonju, South Korea

**Keywords:** weight gain, food craving, schizophrenia, First-Episode Psychosis (FEP), stress, depression

## Abstract

**Objectives:** Food cravings may cause weight gain in patients with schizophrenia. This study investigated psychological characteristics associated with food cravings in patients with first-episode psychosis.

**Methods:** This study analyzed data from a clinical cohort of first-episode psychosis patients taking antipsychotics for 3 months or less. The strength of food cravings was measured using the General Food Cravings Questionnaire-Trait (G-FCQ-T). Psychological characteristics and psychiatric symptoms were investigated with the Positive and Negative Symptom Scale (PANSS), Calgary Depression Scale for Schizophrenia (CDSS), Social and Occupational Functioning Assessment Scale, Rosenberg Self-Esteem Scale (RSES), and Perceived Stress Scale (PSS). Clinical characteristics were compared according to significant weight gain (≥10% increase in body weight compared to baseline) over 3 months. Associations between the G-FCQ-T and other psychiatric scales were investigated. We conducted sex-stratified analyses.

**Results:** In total, 182 patients (78 males and 104 females) with first-episode psychosis were enrolled in this study. In females, the G-FCQ-T total score at baseline was associated with baseline body weight and significant weight gain over 3 months. The PSS scales were significantly associated the G-FCQ-T total and all subscale scores in female participants. Scores on the RSES and CDSS were significantly associated with the G-FCQ-T total score and with the preoccupation and loss of control subscale scores. The PANSS negative and general subscales were significantly associated with the positive outcome expectancy and loss of control subscales of the G-FCQ-T, respectively. In males, the only significant association was between the loss of control subscale and RSES scores. Linear regression analysis showed significant associations of PSS scores with the total and all subscale scores of the G-FCQ-T despite the loss of significance for other variables.

**Conclusion:** These results indicate that the food cravings in patients with first-episode psychosis, which were associated with weight gain, were influenced by perceived stress in females. To reduce food cravings in female patients with schizophrenia, interventions aimed at perceived stress should be considered.

## Introduction

The relative risk of obesity in patients with schizophrenia is 1.5–2 times higher than that in the general population (1). Patients with schizophrenia also have higher rates of diabetes, hypertension, and hyperlipidemia (1–3). A meta-analysis of 77 publications found that the prevalence of metabolic syndrome in patients with schizophrenia was 32.5% (4). This rate is quite high compared to that in the general population; the International Diabetes Federation (IDF) estimated that the global prevalence of metabolic syndrome was 25% (5). In Korea, the prevalence of metabolic syndrome in patients with schizophrenia was reported to be higher (35–43%) (6–8) than that in the general population (20.3%) (9). These chronic physical diseases and metabolic syndrome can adversely impact mortality and general health.

Maintenance treatment with antipsychotics is essential for patients with schizophrenia, although those who are also obese are twice as likely to discontinue using their medication (10). Several studies have also indicated that high body mass index (BMI) (11, 12) and weight gain (13) are associated with a poor quality of life in patients with schizophrenia. Weight gain in patients with schizophrenia is a major public health issue associated with significant health and economic costs (14). There is growing recognition of the need for interventions to address obesity and weight gain in patients with schizophrenia (15–18). It is critical to explore the factors that lead to weight gain in this population in detail.

Antipsychotic treatment has a significant effect on weight gain (19–21). However, several groups have suggested that schizophrenic patients are liable to develop metabolic abnormalities even in the absence of antipsychotic medication (22, 23). Unhealthy eating habits, such as consuming instant meals instead of fresh groceries, have been reported in patients with schizophrenia (24). It remains uncertain whether antipsychotics cause weight gain directly, by decreasing metabolism, or indirectly via increased appetite and decreased activity (25). Therefore, non-drug factors are also important to consider with respect to weight gain in patients with schizophrenia.

In the general population, certain eating habits, food cravings, and psychological factors are associated with body weight and weight gain (26, 27). High levels of perceived stress are associated with a poor diet (28), increased consumption of snack foods (29, 30), decreased consumption of fruit (29, 30), binge eating (31), and increased disinhibition (32), all of which can lead to weight gain. Depression and anxiety have also been shown to increase cravings for palatable foods (33).

Most previous studies on weight gain in patients with schizophrenia have focused on biological factors, including the type of antipsychotics used, rather than on psychosocial and behavioral factors. In addition, weight gain in patients with schizophrenia occurs early in the disease course (34). However, many studies on the eating habits of patients with schizophrenia have been conducted in chronic patients, and the potential confounding effect of the type of antipsychotic medication has not been ruled out.

This study hypothesized that (i) food cravings are associated with weight gain in patients with first-episode psychosis, and (ii) various psychological factors can contribute to food cravings. To minimize confounding factors, only patients with first-episode psychosis, and those using antipsychotics with a low to moderate propensity to cause gain weight, were included in this study. In addition, we conducted sex-stratified analyses to account for possible sex differences in eating patterns.

## Methods

### Study Design

This study analyzed the data of an early-psychosis cohort enrolled in the Gwangju Early Treatment and Intervention Team (GETIT) study (35). The GETIT cohort included patients with a duration of treatment for psychotic symptoms of ≤2 years who met the criteria for “Schizophrenia Spectrum Disorder and Other Psychotic Disorders,” according to the Diagnostic and Statistical Manual of Mental Disorders, Fifth Edition (DSM-5) (36). The patients included in in this study were experiencing first-episode schizophrenia, schizophreniform disorder, or another schizophrenia spectrum disorder. To minimize confounding effects of the type or duration of antipsychotic medication, only patients who took aripiprazole, amisulpride, or paliperidone (risperidone) for 3 months or less were included. Patients with a substance- or medication-induced psychotic disorder, psychotic disorder due to another medical condition, or severe neurological or medical disorder were excluded. This study was conducted from September 2015 to December 2019 and was approved by the Chonnam National University Hospital Institutional Review Board. All subjects provided written informed consent before participation.

### Study Population and Measures

Baseline sociodemographic and clinical data included age, sex, diagnosis, type of antipsychotics taken, dosage of chlorpromazine equivalent dosage (37), duration of treatment, and the duration of untreated psychosis (DUP), which was defined as the time between the appearance of the first psychotic symptoms and the start of antipsychotic treatment (38). Body weight was measured at the start of the study and after 3 months.

The strength of food cravings was measured using the General Food Cravings Questionnaire-Trait (G-FCQ-T), which is a 21-item self-report measure of the general “desire for food” or “desire to eat.” (39) and formally standardized in Korean version (40). The General Food Cravings Questionnaire (G-FCQ), a modified version of the Food Craving Questionnaire (FCQ) (41), was developed by Nijs et al. (39) and consists of two subscales: the G-FCQ-T and General Food Cravings Questionnaires-State (G-FCQ-S). The G-FCQ-T measures the frequency and intensity of general food cravings, while the G-FCQ-S measures the intensity of momentary food cravings. The G-FCQ-T consists of four subscales, and includes six items on preoccupation with food (i.e., obsessively thinking about food and eating), six items on loss of control (i.e., the tendency toward disinhibited eating behavior when exposed to food cues), five items on positive outcome expectancy (i.e., believing eating to be positively or negatively reinforcing), and four items on emotional craving (i.e., the tendency to crave food when experiencing negative emotions). Each item was scored on a six-point Likert scale (1 = strongly disagree, 6 = strongly agree).

Other psychological characteristics and psychiatric symptoms were investigated using the Positive and Negative Syndrome Scale (PANSS) (42), Calgary Depression Scale for Schizophrenia (CDSS) (43), Social and Occupational Functioning Assessment Scale (SOFAS) (44), Rosenberg Self-Esteem Scale (RSES) (45), and Perceived Stress Scale (PSS) (46). All psychiatric scales were completed at baseline.

### Statistical Analysis

Comparisons of clinical characteristics according to “clinically significant weight gain,” defined as a ≥10% increase in body weight after 3 months compared to baseline, were conducted using the chi-square test for categorical variables, independent *t*-tests for normally distributed variables (tested by the Kolmogorov-Smirnov test), and Mann-Whitney U tests for non-normally distributed variables, as appropriate. All clinical scales submitted to statistical analyses were measured at baseline. A two-way analysis of variance (ANOVA) with interactions was conducted to compare participants' clinical scale scores according to sex and significant weight gain. Then, statistical analyses were separately conducted in males and females. Scores on the G-FCQ-T and psychiatric scales were compared between the two groups divided by significant weight gain using an analysis of covariance after controlling for baseline body weight. Associations between G-FCQ-T scores and psychiatric scales were investigated using Pearson's correlation test for normally distributed variables and Spearman's correlation test for non-normally distributed variables. Age and variables that were significantly associated with G-FCQ-T scores at the *p* <0.05 level were entered into linear regression analysis to control for confounding effects. Non-normally distributed variables were entered into the regression model after log transformation. The sample size calculated using GPower for a correlation test with a power of 0.80 and an alpha of 0.05 for a medium effect size (0.3) was 84 for each sex. All statistical tests were two-tailed and *p* < 0.05 was taken to indicate statistical significance. The statistical analysis was performed using SPSS software (version 25.0; IBM Corp., Armonk, NY, USA).

## Results

A total of 182 patients (78 males and 104 females) with first-episode psychosis were enrolled in this study. The media (interquartile range) age at baseline was 25.5 (21.0–32.0) years. The median (interquartile range) duration of treatment and DUP were 1 (0.6–1.5) and 3 (1–12) months, respectively. There were no significant sex differences in these variables. The scores on the G-FCQ-T did not differ significantly between sexes. The scores on the emotional craving subscale of the G-FCQ-T tended to be higher in females than in males, but the difference was not statistically significant (*t* = −1.891, *p* = 0.060). Aripiprazole, amisulpride, and paliperidone were prescribed in 36 (19.8%), 57 (31.3%), and 89 patients (48.9%), respectively. Scores on the G-FCQ-T were not significantly associated with type and chlorpromazine equivalent dosage of antipsychotic medication (all *p* > 0.1, data not shown).

Pearson correlation coefficients between the G-FCQ-T scores and baseline body weight were significant only for the loss of control subscale and total scores in female patients (*r* = 0.346 and 0.251, *p* = 0.002 and 0.013, respectively). A total of 150 patients (82.4%) completed the 3-month follow-up evaluation of weight gain. Clinically significant weigh gain was found in 44 patients (29.3%). Table 1 shows demographic and clinical characteristics, including G-FCQ-T scores, according to the clinically significant weight gain status in the total population. No significant group difference in age, sex, DUP, duration of treatment, or type of antipsychotics and their chlorpromazine equivalent dosage were observed. Scores on the preoccupation subscale score of the G-FCQ-T, the PANSS general and total, and the SOFAS were significantly higher in the group with significant weight gain.

**Table 1:** Demographic and clinical characteristics according to clinically significant weight gain status.

	**Weight gain ≥10% (*n* = 44, 29.3%)**	**Weight gain <10% (*n* = 106, 70.7%)**	Statistical value^*^	***p*-value**
Sex, *n* (%):				
Male	22 (32.8)	45 (67.2)	0.717	0.397
Female	22 (26.5)	61 (73.5)		
Age, years, med (IQR)	24.5 (21.0–30.0)	25.0 (20.0–32.0)	−0.409	0.682
Duration of Untreated Psychosisd, months, med (IQR)	2.0 (1.0–9.5)	2.6 (1.0–15.5)	−0.335	0.738
Duration of treatment, months, med (IQR)	1.0 (0.6–1.0)	1.0 (0.6–1.5)	−1.259	0.208
Type of Antipsychotics, *n* (%)			0.352	0.839
Aripiprazole	8 (28.6)	20 (71.4)		
Amisulpride	15 (32.6)	31 (67.4)		
Paliperidone	21 (27.6)	55 (72.4)		
Bodyweight at baseline, kg, M (SD)	59.6 (10.1)	62.8 (12.1)	−1.565	0.120
Bodyweight at 3 months, kg, M (SD)	67.7 (10.5)	64.6 (12.4)	1.434	0.154
Weight gain for 3 months, kg, M (SD)	8.1 (1.7)	1.8 (2.6)	17.512	**<0.001**
CPZ Eq. dosage, mg/day, med (IQR)	345 (200–630)	400 (200–600)	−0.717	0.473
General Food Cravings Questionnaires-Trait, M (SD)				
Preoccupation	15.9 (6.5)	13.4 (6.1)	2.201	**0.029**
Loss of control	18.3 (6.2)	17.0 (6.9)	1.137	0.257
Emotional craving	13.3 (4.7)	11.7 (5.2)	1.780	0.077
Positive outcome expectancy	17.9 (5.1)	17.1 (5.5)	0.826	0.410
Total	65.4 (19.8)	59.2 (20.7)	1.704	0.090
Positive And Negative Syndrome Scale, M (SD)				
Positive	16.3 (5.1)	15.4 (4.7)	1.009	0.314
Negative	17.9 (4.9)	16.4 (4.3)	1.856	0.066
General	37.1 (8.0)	33.6 (7.2)	2.604	**0.010**
Total	71.3 (15.3)	65.5 (14.2)	2.233	**0.027**
Calgary Depression Scale for Schizophrenia, med (IQR)	3.0 (0.3–8.0)	4.0 (1.0–8.0)	−0.945	0.345
Social and Occupational Functioning Assessment Scale, M (SD)	56.5 (11.0)	60.5 (8.8)	−2.319	**0.022**
Perceived Stress Scale, M (SD)	21.2 (5.8)	20.1 (6.9)	0.918	0.360
Rosenberg Self-Esteem Scale, M (SD)	22.0 (6.9)	21.9 (3.0)	−0.211	0.833

* *Statistical tests are chi-square test, independent t-tests, or Mann-Whitney U tests, respectively. Statistical values are T, Z, or χ^2^, respectively. Values in bold show statistical significance*.

*M, mean; SD, standard deviation; med, median; IQR, Interquartile range; CPZ Eq., chlorpromazine equivalent*.

A two-way ANOVA revealed no significant sex × weight gain interaction except for the RSES (*F* = 4.582, *p* = 0.034). The RSES score was lower in the weight gain group than in the non-weight gain group for females, and it was higher in the weight grain group than the non-weight gain group for males; the results of separate analyses in each sex were not statistically significant (Table 2). Table 2 shows comparisons of psychiatric scale scores according to clinically significant weight gain status for each sex, adjusted for baseline body weight. In female patients, the total G-FCQ-T score and the preoccupation and loss of control subscale scores were significantly higher in the group with significant weight gain. The SOFAS score was significantly lower in patients with significant weight gain.

**Table 2:** Comparisons of psychiatric scale scores according to significant weight gain status for each sex.

	**Female**			**Male**		
	**Weight gain ≥10% (*n* = 22)**	**Weight gain <10% (*n* = 61)**	p^*^	**Weight gain ≥10% (*n* = 22)**	**Weight gain <10% (*n* = 45)**	p^*^
	**M (SD)**	**M (SD)**		**M (SD)**	**M (SD)**	
General Food Cravings Questionnaires-Trait						
Preoccupation	17.5 (6.3)	13.7 (6.2)	**0.013**	14.4 (6.5)	13.1 (6.1)	0.365
Loss of control	20.0 (5.2)	16.9 (6.9)	**0.027**	16.7 (6.9)	17.1 (7.0)	0.890
Emotional craving	14.1 (4.0)	12.3 (5.4)	0.140	12.5 (5.2)	10.9 (4.9)	0.188
Positive outcome expectancy	18.5 (5.0)	17.4 (5.3)	0.365	17.3 (5.1)	16.7 (5.9)	0.624
Total	70.0 (17.3)	60.2 (20.8)	**0.035**	60.9 (21.3)	57.7 (20.8)	0.445
Positive And Negative Syndrome Scale						
Positive	16.3 (5.8)	15.7 (5.1)	0.688	16.4 (4.3)	15.2 (4.2)	0.277
Negative	17.8 (5.3)	16.3 (3.8)	0.180	18.0 (4.6)	16.7 (4.9)	0.707
General	37.9 (9.2)	34.1 (6.7)	0.055	36.3 (6.8)	33.0 (7.7)	0.151
Total	72.0 (18.3)	66.0 (13.6)	0.139	70.7 (12.1)	64.8 (15.2)	0.229
Calgary Depression Scale for Schizophrenia	4.9 (4.9)	4.6 (3.7)	0.686	3.7 (3.6)	5.1 (4.2)	0.274
Social and Occupational Functioning Assessment Scale	54.5 (12.6)	61.0 (8.3)	**0.012**	58.6 (8.9)	59.8 (9.5)	0.851
Perceived Stress Scale	22.5 (3.1)	21.8 (2.9)	0.096	19.9 (5.5)	20.9 (6.6)	0.577
Rosenberg Self-Esteem Scale	25.7 (5.3)	28.0 (5.6)	0.096	27.3 (5.4)	25.0 (6.4)	0.149

* *Adjusted for baseline body weight. All values of scales are described as mean (standard deviation). Values in bold show statistical significance*.

*M, mean; SD, standard deviation; CDSS, Calgary Depression Scale for Schizophrenia; SOFAS, Social and Occupational Functioning Assessment Scale*.

Spearman correlation analysis showed that the G-FCQ-T total score was significantly associated with age in the female population (*r* = −0.222, *p* = 0.023). Spearman correlation analyses showed no significant associations among G-FCQ-T scores, DUP, and duration of treatment (all *p* > 0.1, data not shown). Table 3 shows Pearson or Spearman correlation coefficients between G-FCQ-T scores and clinical variables at baseline. In males, a significant association was only found between the loss of control subscale and RSES scores. In females, the PSS scales were significantly associated the G-FCQ-T total and all subscale scores. Scores on the RSES and CDSS were significantly associated with the total G-FCQ-T scale score and with the preoccupation and loss of control subscales. The PANSS negative and general subscales were significantly associated with the positive outcome expectancy and loss of control subscales on the G-FCQ-T, respectively.

**Table 3:** Correlation coefficients between the General Food Cravings Questionnaires-Trait scores and clinical characteristics for each sex.

	**PANSS_P**	**PANSS_N**	**PANSS_G**	**PANSS_T**	**CDSS**	**SOFAS**	**PSS**	**RSES**
**Female**								
Preoccupation	0.029	−0.049	0.191	0.094	0.208^*^	−0.163	0.346^***^	−0.212^*^
Loss of control	0.119	−0.008	0.259^**^	0.171	0.288^**^	−0.154	0.400^***^	−0.274^**^
Emotional craving	−0.111	−0.189	0.016	−0.083	0.108	−0.053	0.323^**^	−0.061
Positive outcome expectancy	−0.006	−0.209^*^	0.012	−0.055	0.119	−0.065	0.310^**^	−0.089
Total	0.019	−0.119	0.154	0.051	0.220^*^	−0.134	0.405^***^	−0.197^*^
**Male**								
Preoccupation	0.065	0.157	0.120	0.134	0.012	−0.011	0.145	−0.189
Loss of control	0.030	0.022	0.022	0.028	0.084	0.054	0.123	−0.256^*^
Emotional craving	0.018	−0.038	0.138	0.067	0.117	−0.042	0.056	−0.140
Positive outcome expectancy	−0.046	−0.032	0.029	−0.009	0.220	0.067	0.050	−0.145
Total	0.022	0.037	0.085	0.064	0.109	0.022	0.112	−0.215

*Values are Pearson correlation coefficients except those for the CDSS, which are Spearman correlation coefficients. Values in bold show statistical significance*.

* p-value < 0.05;

** p-value < 0.01;

*** *p-value < 0.001*.

*PANSS_P, Positive symptom subscale of Positive And Negative Syndrome Scale; PANSS_N, Negative symptom subscale of PANSS; PANSS_G, General psychopathology subscale of PANSS; PANSS_T, Total score of PANSS; CDSS, Calgary Depression Scale for Schizophrenia; SOFAS, Social and Occupational Functioning Assessment Scale; PSS, Perceived Stress Scale; RSES, Rosenberg Self-Esteem Scale*.

Table 4 shows results of the linear regression analyses in females adjusted for the confounding effects of variables that were significantly associated with the G-FCQ-T in correlation analyses. PSS scores were significantly associated with all subscales and the total score for the G-FCQ-T even after adjusting for other variables. The PANSS negative subscale was significantly inversely associated with positive outcome expectancy on the G-FCQ-T.

**Table 4:** Linear regression analysis to investigate correlates of the General Food Cravings Questionnaires-Trait (G-FCQ-T) after adjusting for age and other psychiatric scales in female population.

	**G-FCQ-T total**		**Preoccupation**		**Loss of control**		**Emotional craving**		**Positive outcome Ex**.	
	**beta**	***p***	**beta**	***p***	**beta**	***p***	**beta**	***p***	**beta**	***p***
Perceived Stress Scale	0.386	**<0.001**	0.318	**0.003**	0.312	**0.003**	0.328	**0.001**	0.352	**<0.001**
RSES	−0.013	0.905	−0.063	0.565	−0.078	0.458	–	–	–	–
CDSS	0.034	0.750	0.009	0.933	0.060	0.581	–	–	–	–
PANSS-Negative	–	–	–	–	–	–	–	–	−0.242	**0.010**
PANSS-General	–	–	–	–	0.080	0.423	–	–	–	–
Age	−0.169	0.066	−0.153	0.106	−0.196	**0.032**	−0.128	0.180	−0.089	0.339
*R*^2^	0.220		0.168		0.247		0.139		0.179	

*RSES, Rosenberg Self-Esteem Scale; CDSS, Calgary Depression Scale for Schizophrenia; PANSS_N, Negative symptom subscale of Positive And Negative Syndrome Scale; PANSS_G, General psychopathology subscale of PANSS; Ex., expectancy*.

*Values in bold show statistical significance*.

## Discussion

Weight gain in patients with schizophrenia begins in the early stage of the disease and rapidly worsens; thus, early intervention is important. In this study, we found that significant weight gain was prevalent in patients who recently started taking antipsychotic medications. Additionally, body weight and weight gain during treatment were significantly associated with food cravings in female patients with first-episode psychosis, but not in males. Associations between food cravings and psychological variables, such as perceived stress, were also prominent in females. There was no significant difference in the degree of food cravings or weight gain according to the type and dosage of antipsychotic drug being taken. To reduce food cravings and uncontrolled eating in patients with schizophrenia, and particularly in females, interventions aimed at perceived stress should be considered. To our knowledge, this is the first study to identify sex-specific associations among food craving, weight gain, and psychological factors in patients with first-episode psychosis.

This study results were consistent with previous studies on the general population showing that food cravings were associated with body weight (47) and weight gain (26). In this study, food cravings were associated with significant weight gain at the 3-month follow-up, in the female participants. Considering that the participants had a median length of 1 month and a median DUP of 3 months, baseline body weight was also likely influenced by weight gain after treatment and food cravings to enrolment in the study.

Sex differences in eating patterns have been noted in the general population, with women usually showing higher levels of dietary restraint and disinhibition than men (48). In addition, women usually partake in more comfort eating than men (49). Women have been found to be more likely to have more food cravings (50), and greater food consumption (51), appetite (52) and weight gain (52), in association with negative emotions. Among our patients with schizophrenia, the females were also more likely to show an increase in food cravings due to psychological factors such as perceived stress.

The stress response is generated by activity in two interacting pathways: the sympathetic adrenal medullary system, in which the release of catecholamines is typical during periods of acute stress (53), and the hypothalamic–pituitary–adrenal (HPA) axis. Acute stress-related sympathetic arousal and glucocorticoid release promote fight-or-flight reactions, which support energy mobilization and gluconeogenesis. Stress-related sympathetic arousal results in redirection of blood flow from the gastrointestinal tract to skeletal muscle and the brain (54). Under an acute stress reaction, suppression of appetite and food intake may occur (55, 56). However, repeated and uncontrollable stress can lead to dysregulation of the HPA axis, which, if chronically activated, can alter glucose metabolism, promote insulin resistance, and influence multiple appetite-related hormones and hypothalamic neuropeptides (57, 58). Moreover, individuals under chronic stress tend to eat more under acute stress conditions and show consume more hyperpalatable, energy-dense “comfort food.” (55, 59) These comfort foods can alleviate stress-related negative affect, as a form of self-medication, via inhibition of hypothalamic corticotropin-releasing factor (CRF) secretion (58, 60), or via temporary normalization of the dopaminergic reward circuit (61–63). Some previous studies have reported correlations between a high level of perceived stress, comfort eating, and decreased HPA activity (64, 65). Repeated stimulation of the reward pathways by palatable foods may lead to neurobiological adaptations that eventually increase food intake in association with food cravings (66–68).

Moreover, depressive symptoms, usually accompanied by chronic stress, are associated with a preference for snack/fast food and sweet food (30, 69), and food addiction (70). Increased intake of convenience food and physical inactivity, accompanied by negative emotional states, are predisposing factors for weight gain (71–73). A meta-analysis of 15 studies (74) showed that depression increased the risk of obesity by 58%. In this study, perceived stress and depression in female patients with first-episode psychosis was associated with food cravings, which could lead to weight gain. However, the effect of depression was not significant in regression analyses, although perceived stress was, suggesting mediating effects of stress on the association between depression and food cravings. The effects of perceived stress and concurrent negative mood on food cravings seen in the general population may also apply to females with early psychosis.

In this study, negative symptoms were inversely associated with positive outcome expectancy relative to food cravings. This finding indicates that negative symptoms such as avolition and anhedonia might inversely affect food cravings in a manner opposite the positive associations found between food cravings and emotional factors such as perceived stress and depression.

Low self-esteem and negative affect have frequently been hypothesized as key features of binge eating, including in the cognitive-behavioral model of bulimia nervosa (75), escape theory (76), emotional regulation theory (77), and schematic models of binge eating (78). These theories posit that individuals showing high self-awareness and negative self-evaluation eventually experience emotional distress, including anxiety and depressed mood, and use binge eating to try and resolve these emotional problems. This study on the relationships among depressive mood, perceived stress, and food craving suggests that these cognitive structures are also present in patients with first-episode psychosis. Furthermore, an association of low self-esteem with uncontrolled eating was seen. Uncontrolled eating has been associated with reward sensitivity and poor cognitive control, which are associated with impulsivity and maladaptive behaviors (79, 80). These traits might also mediate low self-esteem in patients with early psychosis.

This study had a few limitations. First, in this study, patients taking antipsychotics with a low to moderate propensity to cause weight gain (81) were selected, to control the influence of type of antipsychotic on weight gain and food cravings. Although the effect of antipsychotic type on food cravings was controlled, caution is required when generalizing the present findings to patients taking other types of antipsychotics associated with greater weight gain. Second, food intake was measured subjectively. Nevertheless, we found that weight gain, which frequently occurs in patients with schizophrenia, was affected not only by the type of antipsychotic, but also by self-reported food cravings. Finally, cultural differences should also be considered when assessing the association between food cravings and weight gain in Western patients with schizophrenia.

Our study suggests that psychological mechanisms mediating weight gain in patients with schizophrenia may differ according to sex. Food cravings associated with weight gain in female patients with first-episode psychosis were influenced by emotional factors such as perceived stress. Interventions targeting perceived stress may be necessary to promote control of eating behaviors. Psychological factors and eating habits merit more attention, particularly in female patients with schizophrenia, to prevent weight gain. We believe that our findings can contribute to the development of effective interventions to prevent weight gain in patients with schizophrenia.

## Data Availability Statement

The raw data supporting the conclusions of this article will be made available by the authors, without undue reservation.

## Ethics Statement

The studies involving human participants were reviewed and approved by Chonnam National University Hospital Institutional Review Board. The patients/participants provided their written informed consent to participate in this study.

## Author Contributions

Y-HK, MK, MJ, and S-WK have contributed to the conception and design of the study. S-WK and Y-CC organized the database. S-WK performed the statistical analysis. Y-HK wrote the first draft of the manuscript. SR, H-JN, J-YL, J-MK, and MS critically revised the draft. All authors read and approved the submitted version.

## Conflict of Interest

The authors declare that the research was conducted in the absence of any commercial or financial relationships that could be construed as a potential conflict of interest.
